# Granulomatous Inflammation in ANCA-Associated Vasculitis

**DOI:** 10.3390/ijms22126474

**Published:** 2021-06-17

**Authors:** Antje Müller, Bettina Krause, Anja Kerstein-Stähle, Sara Comdühr, Sebastian Klapa, Sebastian Ullrich, Konstanze Holl-Ulrich, Peter Lamprecht

**Affiliations:** 1 Department of Rheumatology & Clinical Immunology, University of Luebeck, 23562 Luebeck, Germany; bet.krause@uke.de (B.K.); anja.kerstein@uksh.de (A.K.-S.); sara.comduehr@uksh.de (S.C.); sebastian.klapa@uksh.de (S.K.); peter.lamprecht@uksh.de (P.L.); 2 Institute of Anatomy & Experimental Morphology, University Hospital Hamburg-Eppendorf, University of Hamburg, 20251 Hamburg, Germany; sebastian.ullrich@krankenhaus-kiel.de; 3 Institute of Experimental Medicine c/o German Naval Medical Institute, Carl-Albrechts University of Kiel, 24119 Kronshagen, Germany; 4 Municipal Hospital Kiel, 24116 Kiel, Germany; 5 Pathologie Hamburg, Labor Lademannbogen MVZ GmbH, 22339 Hamburg, Germany; holl-ulrich@pathologie-hamburg.de

**Keywords:** granuloma, granulomatosis with polyangiitis, autoimmune disease, inflammation, destruction, tissue

## Abstract

ANCA-associated vasculitis (AAV) comprises granulomatosis with polyangiitis (GPA), microscopic polyangiitis (MPA), and eosinophilic granulomatosis with polyangiitis (EGPA). While systemic vasculitis is a hallmark of all AAV, GPA is characterized by extravascular granulomatous inflammation, preferentially affecting the respiratory tract. The mechanisms underlying the emergence of neutrophilic microabscesses; the appearance of multinucleated giant cells; and subsequent granuloma formation, finally leading to scarred or destroyed tissue in GPA, are still incompletely understood. This review summarizes findings describing the presence and function of molecules and cells contributing to granulomatous inflammation in the respiratory tract and to renal inflammation observed in GPA. In addition, factors affecting or promoting the development of granulomatous inflammation such as microbial infections, the nasal microbiome, and the release of damage-associated molecular patterns (DAMP) are discussed. Further, on the basis of numerous results, we argue that, in situ, various ways of exposure linked with a high number of infiltrating proteinase 3 (PR3)- and myeloperoxidase (MPO)-expressing leukocytes lower the threshold for the presentation of an altered PR3 and possibly also of MPO, provoking the local development of ANCA autoimmune responses, aided by the formation of ectopic lymphoid structures. Although extravascular granulomatous inflammation is unique to GPA, similar molecular and cellular patterns can be found in both the respiratory tract and kidney tissue of GPA and MPA patients; for example, the antimicrobial peptide LL37, CD163^+^ macrophages, or regulatory T cells. Therefore, we postulate that granulomatous inflammation in GPA or PR3-AAV is intertwined with autoimmune and destructive mechanisms also seen at other sites.

## 1. Introduction

### ANCA-Associated Vasculitis and Granulomatosis with Polyangiitis and Granulomatous Inflammation

ANCA-associated vasculitis (AAV) comprises three different disease entities, in particular granulomatosis with polyangiitis (GPA), microscopic polyangiitis (MPA), and eosinophilic granulomatosis with polyangiitis (EGPA) [1,2]. In clinical studies, subgrouping of AAV into PR3-AAV and MPO-AAV, i.e., on the basis of ANCA serotype, was more closely linked with genetic factors, biomarkers, and outcomes than classification into clinicopathological categories, i.e., GPA and MPA [2,3,4]. However, subgrouping of AAV into PR3-AAV and MPO-AAV, as has been suggested, conceptually excludes ANCA-positive and ANCA-negative EGPA, as well as rare cases of ANCA-negative GPA and ANCA-negative MPA [3]. In the majority of cases observed in the Northern and Central European population, the classification into GPA corresponds to PR3-AAV [5,6], but MPO-ANCA-associated GPA is infrequently observed as well [7]. Moreover, a DCVAS-based study showed that ANCA specificities vary with regard to different ethnicities [2,5]. Regarding the granulomatous inflammation, it is assumed that it is frequent in PR3-AAV but nearly absent in MPO-AAV [2]. Additionally, it was shown previously that extravascular granulomatosis differentiates GPA and EGPA from MPA [8,9,10,11]. Further, eosinophilic inflammation segregates EGPA from GPA and MPA, but the role of ANCA (usually MPO-ANCA) in ANCA-positive EGPA remains unclear [12]. GPA or PR3-AAV can manifest as upper and/or lower respiratory tract disease with predominant granulomatous inflammation and apparently less renal involvement [13]. In PR3-AAV, granulomatous inflammation is concomitant with tissue damage [9,14], which is a relevant pathophysiological feature of systemic chronic inflammatory disease in general [15]. Current knowledge assigns local injury in AAV as result of a combination of various risk factors (among them ageing) plus autoreactive elements, leading to a number of events such as autoantigen deposition that finally culminate in endothelial and tissue damage [2]. With respect to GPA or PR3-AAV, this translates into the appearance of extravascular granulomatosis, primarily in the upper and lower respiratory tract, which when full-blown is manifested as a classical histological triad consisting of granulomatous inflammation with geographic necrosis and necrotizing small vessel vasculitis [8,16,17,18,19]. In more detail, extravascular granulomatosis in GPA starts with the emergence of neutrophilic microabscesses demarcated by macrophages and histiocytes, or epithelioid cells, which over time begin to form ill-defined granulomas. Subsequently, this turns into larger areas of geographic necrosis, again demarcated by palisading histiocytes and multinucleated giant cells (MGC) and accompanied by the accumulation of more monocytes and macrophages as well as lymphocytes [8,9,16,17,18]. Furthermore, MGC found in close proximity to neutrophilic microabscesses are considered as primary granulomatous feature and occur in both upper and lower respiratory tract [2,16,20,21,22]. Importantly, neutrophils infiltrating tissue in GPA or PR3-AAV display signs of leukocytoclasia such as karyorrhexis or pyknosis, which indicates the necrotizing character of the microabscess and fosters subsequent granuloma formation [16,17]. Besides the development of necrotizing granulomas, GPA features an ANCA-induced necrotizing vasculitis [8,23]. Generally, the ultimate function of a granuloma is described as containment and eradication of microbes or chronic irritants [24]. However, maybe comparable to the granuloma in sarcoidosis [25], while the granulomatous cellular response itself implies an antigen-induced disease [8,16], the initial antigen-independent insult(s) and/or initiating antigen(s) in GPA still remain obscure. In our opinion, granulomatous inflammation in GPA or PR3-AAV can be considered as a relevant factor in the self-amplification of the pathology by continuously supplying autoantigens such as PR3 and subsequently by building structures that are able to generate autoimmune responses in situ [6,26]. Thus, exaggerated antigen-independent innate immune responses, priming neutrophils positioned in the airway mucosa and resulting in microabscesses and granuloma formation [17,27], and ectopic lymphoid structures that sustain autoimmunity to PR3 [22,28] are inextricably intertwined once GPA or PR3-AAV is established. However, although a lot of progress has been reached in deciphering molecular and cellular functions contributing to the pathogenesis of AAV, the mechanisms underlying the formation of neutrophilic microabscesses with subsequent granuloma formation and, finally, scarred or destroyed tissue and bone in GPA or PR3-AAV are still incompletely understood [2,17,29]. To gain more insight, this review aims at summarizing current knowledge on molecular and cellular patterns present in granulomatous inflammation of the respiratory tract in comparison to inflammation in the kidney in GPA and AAV. Special emphasis will be given to migratory patterns and microenvironmental and endogenous factors as well as to the contribution of innate and adaptive immune responses and mechanisms leading to tissue destruction.

## 2. Affected Tissues in GPA and AAV Indicate Involvement of Local Migratory Patterns and Endothelial Activation

Local inflammation requires the infiltration of leukocytes into tissues and organs and is therefore considered as hallmark step [30,31,32]. In this context, it is well known that chemokines and their receptors control the migratory patterns, positioning, and cellular interactions of immune cells [33]. There are several studies reporting on the expression of such molecules in respiratory tract and renal tissue of GPA or AAV and respective speculations on their potential involvement in the recruitment of immune cells. For instance, IFNγ produced by T lymphocytes as well as RANTES produced by macrophages due to their presence in upper or lower respiratory tract tissue of GPA have been suggested to contribute to immune cell homing [34,35]. Correspondingly, cells expressing a receptor of RANTES, i.e., CCR5^+^ cells, were observed in upper respiratory tract tissue of GPA, indicating their possible involvement as well [36,37]. With respect to B cells, recruitment could be mediated by CXCL13 interacting with CXCR5^+^ B cells, as demonstrated by their expression in renal tissue of patients with ANCA-associated nephritis [38]. Further, the increased number of CXCR3^+^ Tregs found in renal tissue of patients with ANCA-associated glomerulonephritis could control excessive Th1 responses [39], and trafficking may be due to interaction with the ligand CXCL9, but the in situ presence of the latter remains to be shown. In addition, the elevated amount of CXCL5 mRNA found in renal tissue of patients with ANCA-associated glomerulonephritis was linked to an influx of destructive neutrophils according to data from animal experiments [40]. Furthermore, CCL2^+^ cells are found in renal tissue, and urinary CCL2 serves as biomarker in ANCA-associated kidney disease [41,42,43]. It is also well acknowledged that infiltrating leukocytes interacting with the endothelium form the primary step in the cascade of cellular migration into tissue [30,32]. In terms of the involvement of endothelial cells in human tissue, only few studies have been performed. For instance, glomerular endothelial cells as well as peritubular capillaries displayed an increased expression of TLR4, and correlation analyses indicated an association with the severity of renal injury [44]. These findings correspond with data of another study showing that an activation of endothelial NFκ-B in glomeruli of patients with ANCA-associated glomerulonephritis may be due to interaction with neutrophils [45]. Early studies of AAV described an accumulation of dying neutrophils around vessels [14], indicating that infiltration of necrotizing neutrophils affects vessels directly [16]. Moreover, circulating neutrophils of AAV patients display a decreased expression of CXCR1 and CXCR2, which could contribute to vascular damage in situ through extended adhesion of neutrophils to the endothelium [46]. 

## 3. Affected Tissues in GPA and AAV Display Cellular and Molecular Patterns Linked to Microbial Infections and Sterile Inflammation 

Exposure to exogenous pathogenic microbes has been suspected as player in the induction of pathogenic immune responses in AAV. Foremost, nasal carriage of *S. aureus* is prominent in GPA and was shown to be linked to endonasal disease activity and the relapse rate [47,48]. In line with these observations, the transcriptomic profile of circulating CD4^+^, CD8^+^, and CD4^+^CD8^+^ T cells derived from GPA patients featured strong associations between upregulated genes and an *S. aureus* infection [41]. Besides the link to *S. aureus*, there is also evidence that a concomitant infection with viruses such as CMV and EBV is associated with the expansion of CD28^-^ T cells in GPA [49]. CD28^-^ T cells are also found in granulomatous inflammation of the upper respiratory tract [50]. Further, local injury and subsequent inflammation may not only alter the physiology of the affected tissue itself, but also change endogenous factors such as the microbiome, which in turn could modulate the local inflammatory response in AAV. Indeed, according to culture-dependent and culture-independent sequencing methods, dysbiosis and reduced diversity of the nasal microbiome were respectively detected in GPA when compared to healthy controls [51,52,53]. Possibly due to differences in study design, one study observed no difference between GPA and controls with respect to abundance of *S. aureus* [51], while carriage of *S. aureus* was found to be prominent in nasal mucosa of GPA compared to controls in the other studies [52,53]. The latter finding supports the putative link between *S. aureus* colonization in the upper respiratory tract and PR3-ANCA^+^ GPA [47,48]. Studies on the distribution of clonal complexes as well as associations with genetic loci of *S. aureus* also yielded differences between PR3-AAV and MPO-AAV [54,55]. Moreover, on the basis of their data, the authors postulated that a *S. aureus*-provoked inflammation favors the accumulation of neutrophils and subsequent granulomatous inflammation in the upper respiratory tract of GPA [55]. In terms of MPO and *S. aureus*, a liaison with the autoimmune response could even be more direct, since a 19mer peptide found in some strains is homologous with an MPO peptide and thus was designated as inducer of autoimmunity against MPO [56]. 

Because of the unknown cause(s) and apart from potential microbial triggers, sterile inflammation needs to be considered as a trigger of granulomatous inflammation in GPA as well, and thus can be recognized by corresponding endogenous immune-stimulatory patterns [32]. Indeed, altered local mRNA and protein expression of DAMP and their receptors is observed in GPA and AAV (see Table 1) [57,58,59]. Among them is HMGB1, a DAMP that was shown to be released into the cytoplasm and the extracellular space in respiratory tract tissue of GPA [58]. In vitro experiments using the pro-inflammatory form of HMGB1 suggested that signaling mediated by the HMGB1 receptor TLR4 participates in both the translocation of autoantigens in neutrophils and the activation of neutrophils by ANCA [60]. Such a mechanism may take place in renal tissue of patients with AAV where neutrophils and monocytes featured expression of TLR4, TLR2, and TLR9, and as pointed out above, glomerular TLR4 and TLR2 expression was suggested to be involved in endothelial activation [44]. While the use of serum HMGB1 as biomarker is judged differently between GPA and AAV [58,61], locally acting necrosis- and inflammation-derived HMGB1 could support the granulomatous inflammation via recruitment of neutrophils as well as monocytes, dendritic cells, and B cells [62,63], possibly even supporting autoimmune responses, at least in GPA. Moreover, increased amounts of urinary HMGB1 were found to be of help in identifying active glomerulonephritis [64]. A local role of HMGB1 is also conceivable with respect to findings showing that TLR9-expressing B and plasma cells derived from patients with MPO-ANCA vasculitis bind to pro-inflammatory HMGB1, thereby inducing B cell proliferation in vitro [65].

Owing to its pauci-immune nature, complement activation has long been regarded as less important for the development of AAV. However, there is accumulating evidence that the alternative pathway of complement activation as well as complement activation product C5a are involved in pathogenic mechanisms [77]. Indeed, the deposition of both properdin and C3d microscopically detected in about half of renal tissue samples from a large cohort of AAV patients indicates that activation of the alternative pathway takes place in situ [78]. In contrast, no properdin was found in kidney biopsies of a small cohort of patients with AAV using mass spectrometry [79], which may be in line with animal studies where properdin was shown to be dispensable for the induction of MPO-ANCA-associated glomerulonephritis [80].

## 4. Cells, Molecules, and Mechanisms of the Innate Immune Response Fuel the Granulomatous Inflammation in GPA 

There is strong evidence that the extravascular granulomatous inflammation predominantly found in PR3-ANCA^+^ GPA or PR3-AAV must be connected to distinct features of the infiltrating cells such as neutrophils or intermediate monocytes exposing a potential autoantigen such as PR3 to the outside tissue microenvironment [17,73,81,82]. Indeed, various properties of circulating neutrophils and monocytes derived from GPA or PR3-AAV patients differ in comparison to healthy neutrophils or to those of MPA or MPO-AAV. For example, a proteomic analysis of circulating neutrophils observed a pattern of 10 proteins distinguishing between GPA and MPA [83]. Importantly, and likely due to genetic reasons, GPA patients exhibit a substantially higher number of circulating neutrophils expressing PR3 on their membrane constitutively [84,85,86]. Consequently, it can be assumed that the amount of PR3 expression at the neutrophil surface is a factor that plays a role for the pathogenicity of antibodies directed against PR3 [87]. In addition, exposure of potential neutrophilic autoantigens such as PR3 occurs as a consequence of mechanisms such as priming for instance mediated by TNFα or C5a, as well as activation and subsequent degranulation of neutrophils [17,88]. Further, cell death mechanisms such as apoptosis, necrosis, and formation of neutrophil extracellular traps (NET) are described as leading to exposure of potential autoantigens [89]. For instance, during neutrophil apoptosis, PR3 is externalized together with other proteins such as phospholipid scramblase 1 and calreticulin [90,91]. Interestingly, in nasal tissue of GPA patients, neutrophils co-expressed PR3 together with cleaved caspase 3, indicating the potential for a local membrane exposure of PR3 through apoptosis [67]. A proteomic analysis of the neutrophilic cytosol discovered intrinsic differences in these proteins (calreticulin, annexin A1, phospholipid scramblase 1) between GPA and healthy controls, pointing towards increased survival and proinflammatory apoptotic neutrophils in GPA [92]. A decreased rate of spontaneous apoptosis of circulating neutrophils in quiescent AAV patients was reported before and it was speculated that this contributes to neutrophil accumulation in regions of inflammation [93]. Of note, expression of another DAMP, S100A9 protein, was found to be increased in the cytosol of apoptotic GPA neutrophils [94] and as part of calprotectin might be useful as serological biomarker in AAV, especially in terms of predicting relapse for PR3-AAV [95,96]. Apart from exposure, a failure of gene silencing mechanisms described for blood neutrophils and monocytes of AAV patients leads to aberrant expression of mRNA for PR3 and MPO [97,98,99], which may contribute to a persistent supply of (auto)antigen and possibly subsequent deposition in situ, i.e., when neutrophils and monocytes migrate into tissue. Additionally, increased PR3 transcription in neutrophil precursors in the bone marrow has been suggested to contribute as well, presumably linked to G-CSF [67]. Furthermore, ageing, a known risk factor of AAV [2], influences neutrophil heterogeneity [100], with aged neutrophils possibly more prone to a pro-inflammatory phenotype or increased NETosis.

The infiltration of neutrophils leading to the appearance of neutrophilic microabscesses in respiratory tract tissue represents an early step of granuloma formation in GPA [8,16,17,18,27]. The formation of neutrophil clusters in interstitial tissue has been designated as “neutrophil swarming” on the basis of a swarm-like migration pattern of neutrophils, which may happen as a consequence of infection or sterile inflammation [101]. Thus, the formation of a neutrophilic cluster such as a microabscess in GPA could also be due to a swarm-like migration pattern. Interestingly, it is thought that coordinated neutrophil swarming acts as seal to prevent influx of microbial pathogens in damaged tissue [102]. Further, Uderhardt and colleagues [103] discovered a previously unknown immune checkpoint in a sterile (laser-induced damage) inflammation that blocks the initiation of neutrophil swarming through so-called “cloaking” of pro-inflammatory debris by resident tissue macrophages. It remains to be evaluated if such resident tissue macrophages are present in granulomatous inflammation of GPA. The “neutrophil swarming” is linked to another physiological defense mechanism that can promote autoantigen exposure, i.e., the aforementioned NET formation or NETosis [104,105]. In fact, when high numbers of neutrophils or neutrophil clusters are present, NET formation occurs in an aggregated fashion [106] that could aggravate a potentially autoantigenic exposure. In terms of the two main ANCA antigens in AAV, NET has been demonstrated as being primarily loaded with perinuclear ANCA antigens such as MPO but not PR3 [107]; thus, for the exposition of PR3 as autoantigen, NET or NETosis can be considered of lesser relevance. This is in line with results demonstrating that NET formation is higher in MPO-ANCA patients compared to PR3-ANCA patients, but it was reported to be largely independent from ANCA [108]. In contrast, it has been shown that antibodies against PR3 and MPO induce NET formation in vitro, and this is controlled by regulated necrosis such as necroptosis of glomerular neutrophils, eventually contributing to MPO-ANCA-mediated glomerulonephritis [109]. Necrosis may also participate directly in the local exposure of potential autoantigens because neutrophils moving towards injured sites in tissue become necrotic [31]. In line with this, the early neutrophil-rich lesion in GPA or AAV is designated as necrotizing, and as mentioned above, the neutrophils show fragmented nuclei as a consequence of apoptosis and necrosis [16,17]. To better understand a potential contribution of necrosis-mediated NET formation or NETosis pathways to autoantigenic exposure in AAV or especially GPA, researchers may need to study neutrophils from AAV or GPA patients because they might behave differently than those of healthy donors, as pointed out previously. The altered properties may then modulate NET formation including exposure of PR3 in GPA or PR3-AAV, and to our knowledge, this has not been investigated thus far. NET formation is described to induce the production of another cytokine, namely, IFNα [110]. A study examining renal tissue from patients with AAV observed a co-localization of two additional DAMP, LL37 and IFNα, in crescentic glomerulonephritis compared to virtually none in renal tissue without crescentic glomerulonephritis. It was proposed that LL37 (maybe as a NET component) mediates the activation of pDC to produce IFNα in the inflamed kidney of AAV patients [111,112]. In line with this observation, a study by Millet and co-workers [67] reported co-expression of PR3 and LL37 accompanied by IFNα expression in respiratory tract tissue of patients with GPA. Thus, the mechanism suggested by Zhang and colleagues [111] could play a role not only in the kidney of AAV patients but in respiratory tract tissue of GPA as well. In addition, a study showed that the amount of circulating pDC is decreased in patients with AAV, and the authors speculated that this is because pDC may have migrated into lymphatic tissue to initiate an autoimmune response [113]. Indeed, dendritic cells are present in both respiratory tract and renal tissue of AAV patients [67,114]. Zhang et al. [111] also stated that the origin of LL37 in the kidney is unclear. Because of the close proximity between PR3 and LL37 in respiratory tract tissue of GPA [67], we speculate that PR3 expressed in abundance by infiltrating neutrophils may be able to cleave hCAP18 into LL37 in situ, i.e., within a microabscess in the tissue. It was demonstrated that the cleavage of hCAP18 by PR3 leading to release of LL37 takes place extracellularly [115], which would imply that for a local presence of LL37, PR3 needs to be expressed either on the cellular membrane or secreted outside the cell. 

Of note, neutrophils from healthy donors secrete preformed macrophage migration inhibitory factor (MIF) during secondary necrosis, and it is speculated that MIF acts as a danger signal for insufficient clearance of apoptotic neutrophils [116,117]. Interestingly, abnormalities of the MIF gene are more frequent among GPA patients, and using a mouse model, it was demonstrated that overexpression of MIF promotes lung granulomas and increased mortality, thus supporting a role for MIF in GPA [118]. Apart from neutrophils and monocytes, NG2^+^ pericytes present around capillaries and arterioles also express MIF [31]. It remains to be determined if NG2^+^ pericytes as well as innate immune cells contribute to local MIF expression or release in GPA. Another molecule that has gained interest with respect to a role in necrotizing vasculitis and the induction of a sterile inflammation is human serum factor H-related protein or FHR1. In particular, it has been shown that FHR1 attaches to necrotic cells in affected kidneys of AAV patients, while this was not the case in healthy renal tissue. In ANCA-associated glomerulonephritis, the immobilization of FHR1 led to a pro-inflammatory cytokine secretion of monocytes mediated by the NLRP3 inflammasome [119]. This fits to a previous study demonstrating IL-1β^+^ cells in renal tissue of AAV [120]. Moreover, it is proposed that phagocytosed viable *S. aureus* in neutrophils via the engagement of RIPK3 trigger the release of serine proteases from cytoplasmic granules with subsequent processing and secretion of IL-1β [121]. In light of the findings mentioned above, such a mechanism seems conceivable in granulomatous inflammation of GPA.

**Figure 1:**
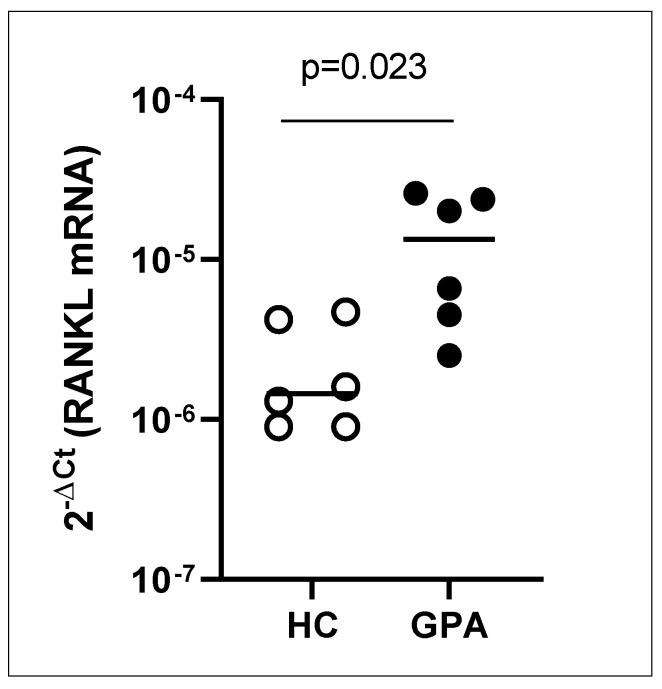
Elevated RANKL mRNA (NM_003701.4) expression by PBMC derived from patients with GPA compared to healthy controls (HC, *n* = 6 each). The 18S rRNA gene (NR_003286.3) was used for normalization. Primer sequences are available upon request.

The granulomatous inflammation of GPA is characterized by findings of a high debris load and overfed macrophages, indicating that an overwhelming production of endogenous tissue debris may act as permanent stimulus for the formation of multinucleated giant cells [20]. Accordingly, TRAP^+^ osteoclast-like multinucleated giant cells in respiratory tract tissue are considered as a characteristic immunopathologic sign of GPA [21]. Moreover, the generation of TRAP^+^ MGC appears to be promoted by members of the TNFSF, among them TNSF11 or RANKL [22,122]. In detail, it was demonstrated that circulating monocytes of patients with generalized GPA display a higher propensity to turn into TRAP^+^ MGC in comparison to healthy controls [122]. In line with this, we observed an increased RANKL mRNA expression by PBMC of GPA patients when compared to healthy controls (Figure 1). Additionally, matricellular proteins such as osteopontin are involved in granuloma formation, especially in the formation of osteoclast-like MGC [123]. In accordance, their gene expressions in nasal mucosa have been shown to differ between GPA and healthy as well as disease controls [59]. Apart from MGC playing roles in the extravascular granulomatosis and tissue destruction, CD163^+^ or M2 macrophages were demonstrated as the predominant type in respiratory tract tissue of GPA [68]. Interestingly, a similar finding was observed in early ANCA-associated glomerulonephritis, where CD163^+^ M2 macrophages were present at sites of both glomerular fibrinoid necrosis and normal-appearing glomeruli [124]. The presence of CD163^+^ macrophages in inflamed tissues of GPA and AAV is in line with findings demonstrating that the efferocytosis of apoptotic neutrophils is mediated by M2 macrophages [125]. A previous study reported relationships between CD68^+^ cells and crescentic glomerulonephritis, necrosis, and serum creatinine in patients with AAV, indicating a role of macrophages in renal tissue destruction [126]. Furthermore, the attraction of CCR8^+^ monocytes by a high number of CCL18^+^ mononuclear or myeloid dendritic cells in renal tissue of patients with ANCA-associated crescentic glomerulonephritis was suggested as a mechanism inducing M2 polarization of circulating monocytes and mediating acute cellular tissue inflammation [127]. In terms of PR3-expressing apoptotic neutrophils in GPA it is assumed that the efferocytosis by macrophages prevents the resolution of inflammation, especially in situ [67,94]. However, it cannot be ruled out that the presence of CD163^+^ macrophages in inflamed tissue of GPA and MPA could also be due to their role in the resolution of inflammation and tissue repair [128,129]. It also needs to be taken into account that a simplified classification according to M1 and M2 subtypes most likely does not reflect the complexity of macrophage polarization in vivo [130,131]. Alternatively, activation-independent core signatures of macrophages may provide a more precise definition [132]. In addition, a study of infectious mouse models demonstrated that alternatively activated macrophages derived from monocytes differ in phenotype and transcriptional profiles from tissue-resident alternatively activated macrophages [133]. The detection of CD163L1^+^ cells and CLEC5A^+^ cells, which may allow a discrimination between resident and monocyte-derived macrophages in vivo, for example in lymphoid tissue [134], might offer a possibility to identify the proportions of such cells in the granulomatous inflammation of GPA or PR3-AAV. While a functionally relevant haplotype of IRF5 detected in GPA was assigned as protective effect [135], in general, IRF5 has been demonstrated to act as an inhibitor of M2 macrophage marker expression [136]. Thus, it might be of interest to find out how the genetic variation of IRF5 in GPA influences the macrophage phenotypes in situ. Altogether, to gain a deeper insight into the spectrum of macrophages in vivo, a comparison of the same tissue under homeostasis and in pathophysiological conditions might be required [132]. In summary, there is substantial evidence that (dysregulated) cell death mechanisms accompanied by release of DAMP and DAMP-induced innate as well as adaptive cellular responses contribute to the loss of tolerance and the development of pathogenic autoimmunity against human PR3 such as in GPA.

## 5. In GPA or PR3-AAV, Granulomatous Inflammation Is a Prerequisite for the Development of Local Autoimmune Responses 

While neutrophil infiltration and microabscess formation dominate the early phase of the granulomatous inflammation in GPA or PR3-AAV, over time, the neutrophil-rich necrotizing lesions are converted into a monocyte/macrophage-rich granuloma, and this is accompanied by infiltration of lymphocytes such as T cells [16,17]. On the basis of various findings (see Table 2) [34,50,69,70,71], it is assumed that lesional T cells belonging to the effector memory type are a source of pro-inflammatory cytokines, support granuloma formation, or act directly on target cells [137]. A detrimental effect of T cells could for instance be exerted by the lack of PD-1 expression of lesional T cells in renal tissue of AAV, which may lead to less regulation of local T cell responses [138]. Otherwise, on the basis of findings showing expression of IL-15 and MICA/MICB, presumably by macrophages and dendritic cells, in both renal and respiratory tract tissue of GPA, researchers have postulated that IL-15 together with MICA/MICB might be able to promote local CD4^+^ T cell proliferation via interaction with NKG2D-expressing cells [66,139]. Because regulatory T and B cells keep unwanted autoimmune responses under control, defects in number and function of these cells can also contribute to autoimmune disease. Accordingly, abnormalities in number and function of Treg cells are present in GPA or PR3-AAV [16]. While CD3^+^FoxP3^+^ T cells have been detected in granulomatous lesions of the respiratory tract in GPA [72,137], it is still unknown if their regulatory functions are impaired in situ, maybe similarly to what has been demonstrated for circulating Treg cells ex vivo [136]. As pointed out above, increased numbers of CXCR3^+^ Treg cells have been found in renal tissue of AAV patients, and on the basis of data from a mouse model, it is thought that these Treg limit an excessive Th1 response [39]. Importantly, renal tissue of patients with ANCA-associated glomerulonephritis featured a substantially higher number of RORγt^+^ CCR6^+^ Th17 cells compared to peripheral blood and controls, and on the basis of animal experiments, researchers suggested that the Th17 cells egress from the intestinal tract [140]. It remains to be determined if Th17 cells are present in respiratory tract tissue of GPA or AAV patients as well. Although regulatory B cells have been investigated in AAV [141,142,143], little if anything is known with regard to their appearance and function in inflamed tissue of GPA or MPA. A study indicated that the number of circulating neutrophils positively correlates with the number of circulating memory B cells in GPA [144]. It may be of interest to find out if this association is maintained in situ as well, especially in light of the increased IL-6 production by circulating CD19^+^ B cells in GPA [144]. Sustained expression of IL-6 has been linked to local perivascular accumulation of B cells and mature plasma cells in a transgenic mouse model [145].

Histologically infiltrating T and B lymphocytes are part of the typical dense lymphoplasmocytic background observed in granulomatous inflammation of GPA [9]. Both respiratory tract and renal tissues of GPA and AAV display lymphocytic infiltrates or follicle-like structures with different levels of organization [22,146]. In more detail, four levels of lymphocytic organization were identified in renal tissue of a large cohort of patients with ANCA-associated glomerulonephritis and a higher degree was associated with renal failure [146]. In general, ectopic or tertiary lymphoid structures (in short ELS or TLS) are found in tissues targeted in several autoimmune diseases such as synovial tissue in rheumatoid arthritis [147,148]. It is thought that TLS develops as a response following expression of pro-inflammatory cytokines, for instance, members of the TNFSF and local cross-talk between inflammatory immune cells and resident stromal cells. It is further argued that these structures fulfill survival needs of aggregated leukocytes (such as neutrophils in inflamed tissue of AAV), which subsequently promotes local adaptive immune responses toward locally displayed antigens (or autoantigens such as structurally altered PR3 or MPO) [148]. Accordingly, it has been proposed that the formation of lymphocytic cell aggregates may promote the presentation of ANCA antigens to T cells and the production of ANCA in situ [2,17,149]. Furthermore, these assumptions support the idea that the maintenance of memory plasma cells in inflamed tissue relies on the same or similar structures and mechanisms, which are found and take place in bone marrow or secondary lymphoid organs [150]. Of note, it was also suggested that TLS evolved prior to secondary lymphoid organs [148,151]. In line with this, APRIL or TNFSF13 is produced and/or secreted locally by neutrophils, macrophages, and MGC in granulomas of GPA and could promote B and plasma cell survival, of which the latter are found in both respiratory tract and kidney tissue of AAV [22,74,75,152]. In terms of local PR3-ANCA production, using immunofluorescence staining with an idiotypic antibody recognizing PR3-ANCA [153], individual PR3-ANCA^+^ B cells could be identified in respiratory tract and renal tissue of GPA patients [28]. For these findings to be validated, the use of labelled enzymatically inactive PR3 employed to detect PR3-specific B cells in peripheral blood of patients with PR3-AAV [154] would be an appropriate alternative. The pathogenicity of antibodies targeting PR3 depends on the targeted (conformational) epitope of PR3 [87,155], and non-pathogenic IgG anti-PR3 antibodies exist, at least in patients with GPA in remission [156]. Immunostaining of human tissues is not sufficient to find out if tissue-resident B or plasma cell in GPA or AAV produce a pathogenic or a non-pathogenic PR3-ANCA, and other methods are thus required. In addition, the finding that remote polymorphisms potentially present in PR3 or remote protein–ligand interactions can lead to the activation and accessibility of a latent epitope recognized by an anti-PR3 antibody derived from a GPA patient [157] points towards high levels of complexity in terms of the relationships between autoantigens and ANCA in GPA and AAV. An intriguing finding has been made with regard to MPO-ANCA vasculitis by demonstrating that CD4^+^ T cells as well as B cells derived from patients with MPO-AAV recognize linear epitopes in a restricted region of human MPO (MPO_447–461_). Moreover, this MPO epitope is hidden physiologically, meaning that for outside exposure of the epitope, the structure of MPO requires substantial alteration, thereby inducing conformational changes [158]. It could be speculated that such a conformational change might be induced locally by inflammatory events in AAV. 

## 6. Granulomatous Inflammation and Tissue Destruction in GPA

**Figure 2:**
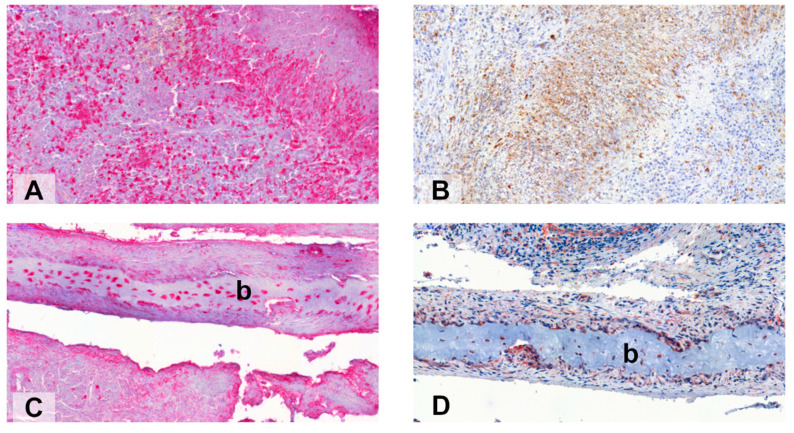
S100A4^+^ cells (red, (**A**)) and CD68^+^ cells (brown, (**B**)) in nasal tissue of GPA (serial staining), presumably palisading histiocytes, marking the edge of a granuloma. S100A4^+^ cells (red, (**C**)) and Vimentin^+^ cells (reddish brown, (**D**)) invading bone (**b**) in nasal tissue of GPA (serial staining), presumably fibroblasts. Immunohistochemistry for S100A4 in nasal tissue of GPA (*n* = 3) was performed as described previously [164]. The upper respiratory tract tissue samples of GPA were characterized in previous studies [22,76].

Apart from classical immune cells, tissue or specialized lymphoid fibroblasts have been acknowledged as major players in chronic inflammatory and autoimmune diseases, for instance rheumatoid arthritis and Sjögren’s syndrome [148,151,159]. However, the mechanisms by which fibroblasts contribute to tissue and bone destruction in GPA or AAV are only partially understood. Inflammatory destruction of bone by pannus-like tissue can be regarded as component of granulomatous inflammation in GPA [9]. Indeed, using a xenograft transplantation model, researchers demonstrated that nasal tissue derived from GPA patients is able to destroy human healthy cartilage to a large extent, mainly mediated by fibroblasts and matrix metalloproteinases (MMP) such as MMP3 and independent of the influx of circulating human cells [76]. Of note, while the activation of resident fibroblasts is considered reliant on leukocytic cytokines as one way of the above-mentioned local cross-talk, a significant lymphocyte migration into tissue is regarded as less important for fibroblast priming [151]. Further, S100A4 or fibroblast-specific protein 1 (FSP-1) is a molecule with cellular expression and release at sites of joint destruction in rheumatoid arthritis, whereby extracellular S100A4 has been shown to increase the expression of MMP-3 [160]. In addition, studies on human and murine pulmonary fibrosis showed that S100A4 is expressed by alternatively activated or CD163^+^ M2 macrophages in the lower respiratory tract [161,162]. In line with these observations, immunohistochemical staining suggests that substantial numbers of S100A4^+^ cells are present in upper respiratory tract tissue of GPA, among them both CD68^+^ macrophages and Vimentin^+^ fibroblasts (Figure 2). Of note, S100A4 has been shown to play a role in the defense against *S. aureus*, whereby a lack of S100A4 led to a more efficient bacterial clearance in a mouse S100A4 knockout model [163]. Thus, cells overexpressing S100A4 in the upper respiratory tract tissue of GPA might contribute to tissue destruction for instance via increased expression of MMP3 by fibroblasts and to granulomatous inflammation possibly via less effective clearance of *S. aureus* promoting neutrophilic accumulation. 

Finally, deposition of collagen and fibrosis in affected tissue, especially of MPO-AAV, are described, but the mechanisms that contribute seem less well investigated [17,27]. Of note, MPO-ANCA IgG has been demonstrated to lead to an increased release of macrophage colony-stimulating factor (M-CSF, CSF-1) in vitro, which subsequently may contribute to fibrosis [165], but it remains to be seen if such a mechanism takes place in vivo as well. 

## 7. Therapeutic Measures and Tissue Damage in AAV

In terms of therapeutic targets, the latest developments center around the inhibition of T and B cells, cytokines, or signaling molecules in AAV, either selectively by employing antibodies such as antibodies targeting CD20, BAFF, or CTLA4, or more general by using plasmapheresis [2,19,166]. Recently, a small molecule inhibitor of complement receptor C5aR1 was introduced as new steroid-sparing therapeutic option in AAV [167], However, it remains to be answered if these therapies also reach affected tissue or organs and for instance dampen the granulomatous inflammation. Despite B cell depletion inflamed tissue might still act as hideaway for autoreactive B lymphocytes in GPA [168]. Thus, further research regarding therapeutic efficacy of the treatments mentioned above in stopping tissue inflammation and destruction in AAV might be required.

## 8. Concluding Remarks

**Figure 3:**
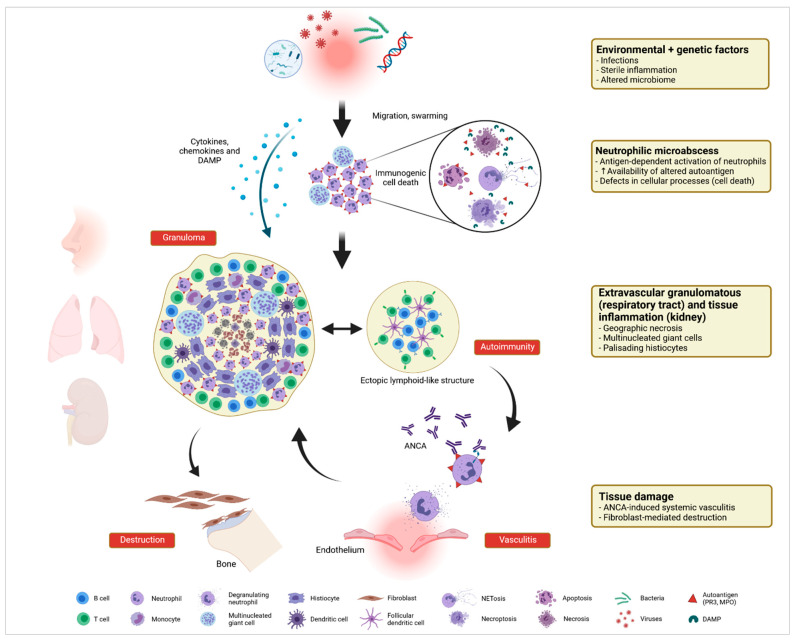
Development and function of the granulomatous inflammation in GPA. A combination of predisposing genetic factors as well as exogenous and endogenous immune-stimulatory patterns such as infections, altered microbiome, and sterile inflammation serve as initial trigger for the recruitment and activation of extravascular primed neutrophils forming neutrophil-rich microabscesses. Appearance of multinucleated giant cells (MGC) in close proximity represents a primary granulomatous feature of this early stage of the granulomatous lesion. Immunogenic cell death (dysregulated apoptosis, necrosis, necroptosis, NETosis) leads to the release of damage-associated molecular patterns (DAMP) and contributes to increased availability of the (altered) autoantigen proteinase 3 (PR3). Concomitant pro-inflammatory cytokines and chemokines drive recruitment of further immune cells, converting the neutrophil-rich necrotizing lesion to a monocyte/macrophage-rich granuloma. Palisading histiocytes around a necrotic zone, accompanied by MGC and the accumulation of monocytes, as well as lymphocytes, define the characteristic histological feature seen in the respiratory tract and the kidney. Occurrence of inflammation-related ectopic lymphoid structures (ELS) set the prerequisite for the loss of tolerance against the autoantigen PR3 that is continuously displayed by high numbers of neutrophils and monocytes within the granulomatous lesion. Tissue damage occurs as result of ANCA-induced systemic vasculitis and inflammation- and fibroblast-mediated local destruction of cartilaginous and osseous tissues in the upper respiratory tract. Created with BioRender.com (accessed on 10 April 2021).

Altogether and focusing on the studies summarized herein, one could imagine that granulomatous inflammation in tissue or organs of GPA or AAV patients is not just an epiphenomenon of ongoing disease but participates by promoting pro-inflammatory and, subsequently, autoimmune and destructive amplification loops in situ (Figure 3). Further, because granulomatous inflammation is more prominent in PR3-ANCA^+^ GPA or PR3-AAV when compared to MPA or MPO-AAV, it can be hypothesized that PR3 is closely linked to the formation of granulomas. On the basis of the findings, one could speculate that, in situ, various ways of exposure linked with a high number of infiltrating neutrophils as well as monocytes lower the threshold for the presentation of a structurally altered PR3 and possibly also of MPO, provoking the development of local autoimmune responses, aided by the formation of ELS, and the destruction of tissue and bone. Because distinct expression patterns of inflammatory molecules and/or cellular structures such as LL37, MICA/MICB^+^ cells, or CD163^+^ macrophages can be found in both respiratory tract and renal tissue of AAV patients, it can be hypothesized that related mechanisms take place, less dependent from the affected site [169]. For more insights into the interplay or overlap between local granulomatous inflammation and the development of autoimmune responses in GPA or PR3-AAV to be obtained, comparative analyses of for instance circulating and tissue-resident leukocytes from the same patient might be desirable.

## Figures and Tables

**Table 1:** Overview about molecular and/or cellular markers of the innate and adaptive immune response suggested to participate in the granulomatous inflammation and to support autoimmune and destructive mechanisms in the upper and lower respiratory tract of GPA patients.

Molecular or Cellular Biomarker	Presence and/or Role in Respiratory Tract	Reference
Anti-microbial peptides, innate immune receptors, cytokines	10 differentially expressed transcripts in the nasal mucosa of GPA patients (*n* = 29)	[57]
gene expression signatures	339 differentially regulated genes in nasal transcriptome of GPA patients (*n* = 32)	[59]
HMGB1	released into the cytoplasm and the extracellular space in endonasal tissue of GPA patients (*n* = 5), marker of granulomatous burden in GPA	[58]
RANTES^+^ cells	RANTES production by macrophages in lung tissue of GPA patients (*n* = 6)	[35]
CCR5+ cells	higher CCR5 expression in nasal tissue of localized GPA patients (*n* = 10)	[36]
CCR5, RANTES	increased number of CCR5^+^ cells in lower respiratory tract tissue of GPA patients (*n* = 4)	[37]
MICA/MICB, IL-15	MICA/MICB-expressing cells and IL-15-expressing cells in nasal tissue of GPA patients (*n* = 5)	[66]
MARCO^+^ Meltrin^+^ macrophages and multinucleated giant cells	necrotic and damaged self-drives granuloma formation in respiratory tract tissue of GPA patients (*n* = 7)	[20]
multinucleated giant cells (MNG)	more MNG in lung compared to nasal tissue of GPA patients (*n* = 23)	[21]
LL37, IFNα, pDC	co-expression of PR3, LL37, and IFNα in respiratory tract tissue of GPA patients (*n* = 6)	[67]
CD163^+^ macrophages	M2 as predominant macrophage type in respiratory tract tissue of GPA patients (*n* = 35)	[68]
IFNγ^+^ cells, TNFα^+^ cells	more IFNγ^+^ CD4^+^ T cells in respiratory tract tissue of localized GPA (*n* = 9 and *n* = 3, respectively)	[34,69]
CD3^+^ T cells, eosinophils, IL-4	more IL-4 expression in nasal tissue of GPA patients (*n* = 10)	[70]
CD28^-^ T cells	accumulation of CD3^+^ CD28^-^ cells in upper respiratory tract tissue of GPA patients (*n* = 5)	[50]
CD3, CD134	CD134^+^ T cells in inflammatory lesions of GPA patients (*n* = 6, respiratory tract)	[71]
CD3, FoxP3	accumulation of Treg cells in upper respiratory tract tissue of GPA patients (*n* = 10)	[72]
CD20, PR3, CD38	follicle-like structures of B cells, plasma cells, and PR3^+^ cells in upper respiratory tract tissue of GPA patients (*n* = 6)	[73]
APRIL, B and plasma cells	mucosal inflammation of GPA is rich in B cell survival factor and autoantigen (*n* = 8)	[74]
IgG4^+^ plasma cells	increased number of IgG4^+^ cells in the head and neck region of GPA patients (*n* = 43)	[75]
Plasma cells, APRIL, ectopic/tertiary lymphoid structures	neutrophils, macrophages, and giant cells contribute to granulomatous inflammation in respiratory tract tissue of GPA patients (*n* = 22)	[22]
PR3-ANCA^+^ B cell	few 5/7 Id^+^/IgG^+^ B cells in inflamed tissue of GPA patients (*n* = 6)	[28]
Fibroblasts, MMP	GPA-related destruction in the upper respiratory tract is mediated by fibroblasts (*n* = 10)	[76]

**Table 2:** Overview about potential molecular and/or cellular markers suggested to participate in inflammatory, autoimmune, and destructive mechanisms in renal tissue of AAV patients (GPA, MPA, or MPO-AAV, respectively, ANCA-associated glomerulonephritis).

Molecular or Cellular Biomarker	Presence and/or Role in Kidney	Reference
MICA/MICB, IL-15	MICA/MICB-expressing cells and IL-15-expressing cells in renal tissue of GPA patients with active glomerulonephritis (*n* = 4)	[139]
LL37, IFNα	co-localization of LL37 and IFNα in renal tissue of AAV with crescentic glomerulonephritis (*n* = 40)	[110]
CCL18+ and CCR8^+^ cells	CCL18+ myeloid dendritic cells, CCR8+ macrophages in ANCA-associated crescentic glomerulonephritis (*n* = 31)	[126]
CXCL5 mRNA	highly upregulated CXCL5 expression in renal tissue of ANCA-associated glomerulonephritis (*n* = 8)	[40]
MCP-1	MCP-1^+^ cells in renal tissue of ANCA-associated glomerulonephritis (*n* = 11)	[41]
Monocytes, neutrophils, T cells	CD68^+^ monocytes/macrophages infiltrating the kidney are linked with tissue damage in AAV (*n* = 65)	[125]
CD163^+^ macrophages	CD163^+^ M2 macrophages are present in glomerular capillaries of ANCA-associated glomerulonephritis (*n* = 17)	[123]
p-MLKL^+^ neutrophils	necroptosis and NET formation contribute to MPO-ANCA-associated glomerulonephritis (*n* = 12)	[108]
NET, LL37	NET deposition found in about half of renal tissue samples of GPA (*n* = 9) and MPA (*n* = 6)	[111]
FHR1	FHR1 marks necrotic-type cells and induces monocytic inflammation in renal tissue of AAV patients (*n* = 79)	[118]
C3d, C4d, C5b-9, properdin	C3d and properdin are associated with cellular crescents in renal tissue of ANCA-associated glomerulonephritis (*n* = 187)	[78]
C3, C4, no properdin	moderate complement activation in kidney tissue of AAV with glomerulonephritis (*n* = 13)	[79]
TNFα^+^ cells, IL-1β^+^ cells, IL-2R^+^ cells	TNFα^+^ cells, IL-1β^+^ cells, and IL-2R^+^ cells are present in renal tissue of AAV patients (*n* = 22)	[119]
IL-2, IL-4	cells expressing IL-2 and cells expressing IL-4 are found in renal tissue of GPA patients (*n* = 10)	[70]
CD208, CD209, CD3	immature dendritic cells cluster with T cells are present in interstitial infiltrates in renal biopsies of AAV patients (*n* = 25)	[113]
PD-1	most lesional T cells lack PD-1 in renal tissue of GPA patients (*n* = 8)	[138]
CXCR3+ Tregs	CXCR3+ Tregs are enriched in the kidney of AAV with crescentic glomerulonephritis (*n* = 8)	[39]
CXCL13, CXCR5	CXCL13+ dendritic cells, CXCR5+ B cells, and intrarenal lymphoid tissue are found in ANCA nephritis (*n* = 16)	[38]
Th17 cells, RORγt	increased number of Th17 cells in the kidney of patients with ANCA-associated glomerulonephritis (*n* = 8)	[140]
Lymphocytic infiltrates	four different levels of lymphocytic organization can be distinguished in renal tissue of ANCA-associated glomerulonephritis (*n* = 122)	[146]
IRF4^+^ cells	plasma cells are present in renal tissue of GPA patients (*n* = 4)	[147]
TLR2^+^ and TLR4^+^ cells	endothelial cells in glomeruli of AAV patients express TLR2 and TLR4 (*n* = 24)	[44]
NFκB p50/p65-expressing endothelial cells	endothelial NFκB is activated in renal tissue of ANCA-associated glomerulonephritis (*n* = 5)	[45]

## Data Availability

Data sharing is not applicable to this article.
